# Correction: Transcriptome Analysis of Resistant and Susceptible Alfalfa Cultivars Infected With Root-Knot Nematode *Meloidogyne incognita*

**DOI:** 10.1371/journal.pone.0123157

**Published:** 2015-03-30

**Authors:** The PLOS ONE Staff

## Notice of Republication

This article was republished on March 11, 2015, to include a missing paragraph in the Results section that was removed in the typesetting process. The originally published, uncorrected article and the republished, corrected article are provided here for reference.

## Supporting Information

S1 File Originally published, uncorrected article. (PDF)

S2 File Republished corrected article. (PDF)
