# Supplementary material for: Transcriptome Analysis of Resistant and Susceptible Alfalfa Cultivars Infected With Root-Knot Nematode Meloidogyne incognita
Source: PLoS One. 2015 Feb 24;10(2):e0118269. doi: 10.1371/journal.pone.0118269 (PMC4339843; doi:10.1371/journal.pone.0118269)
Supplement: S1 Fig — Slide 1. Cluster (A, B) and principal component analyses (C, D) of polyA selection and rRNA depletion cDNA library preparation methods using DESeq2 package. Cv. Lahontan (A, C); cv. Moapa 69 (B, D). Slide 2. Cluster (A) and PCA (B) analysis of Moapa 69 samples with poly A selection type of library preparation using DESeq2 package. (PPTX) [file pone.0118269.s001.pptx]

## Slide 1
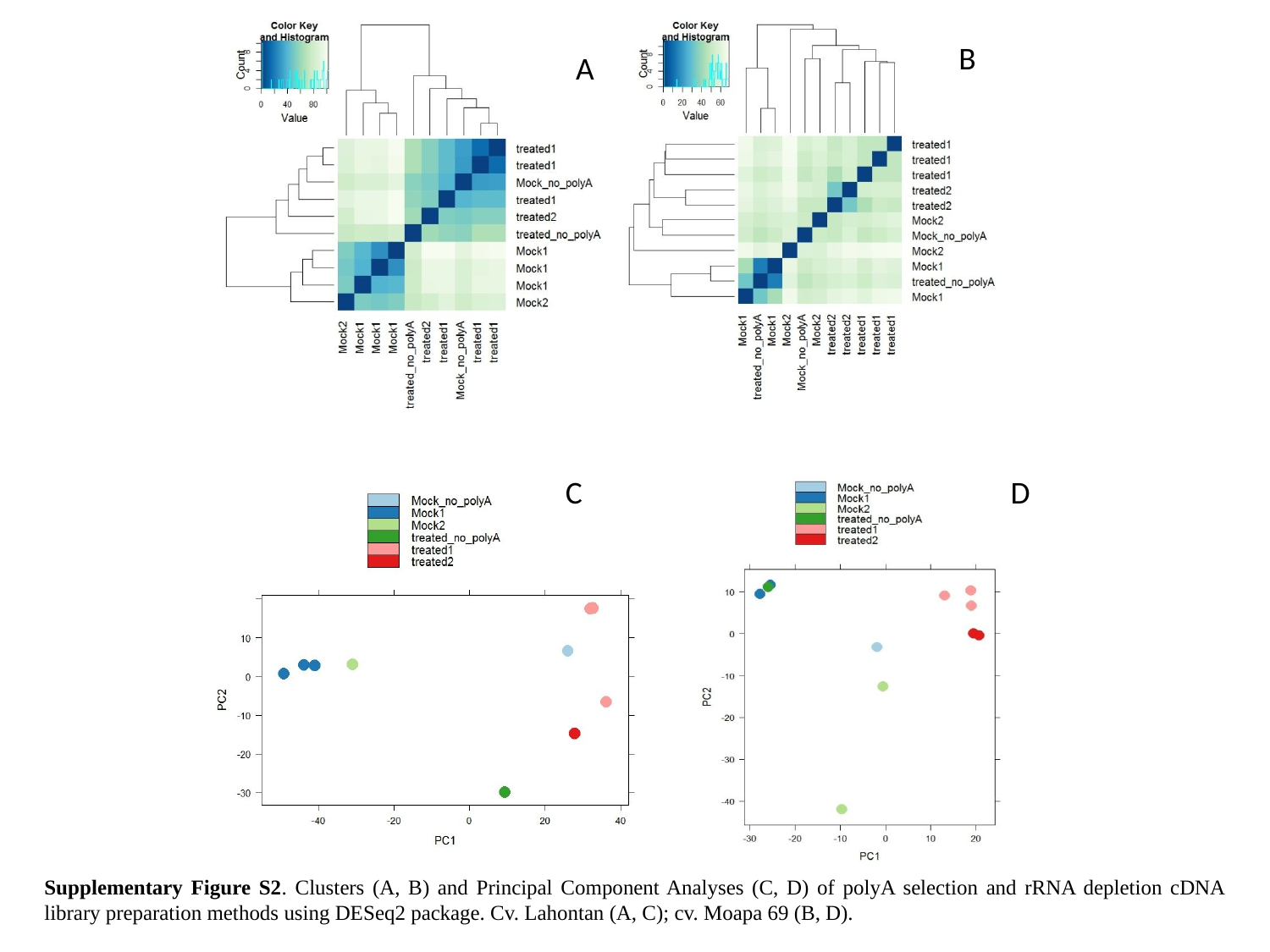

B
A
C
D
Supplementary Figure S2. Clusters (A, B) and Principal Component Analyses (C, D) of polyA selection and rRNA depletion cDNA library preparation methods using DESeq2 package. Cv. Lahontan (A, C); cv. Moapa 69 (B, D).

## Slide 2
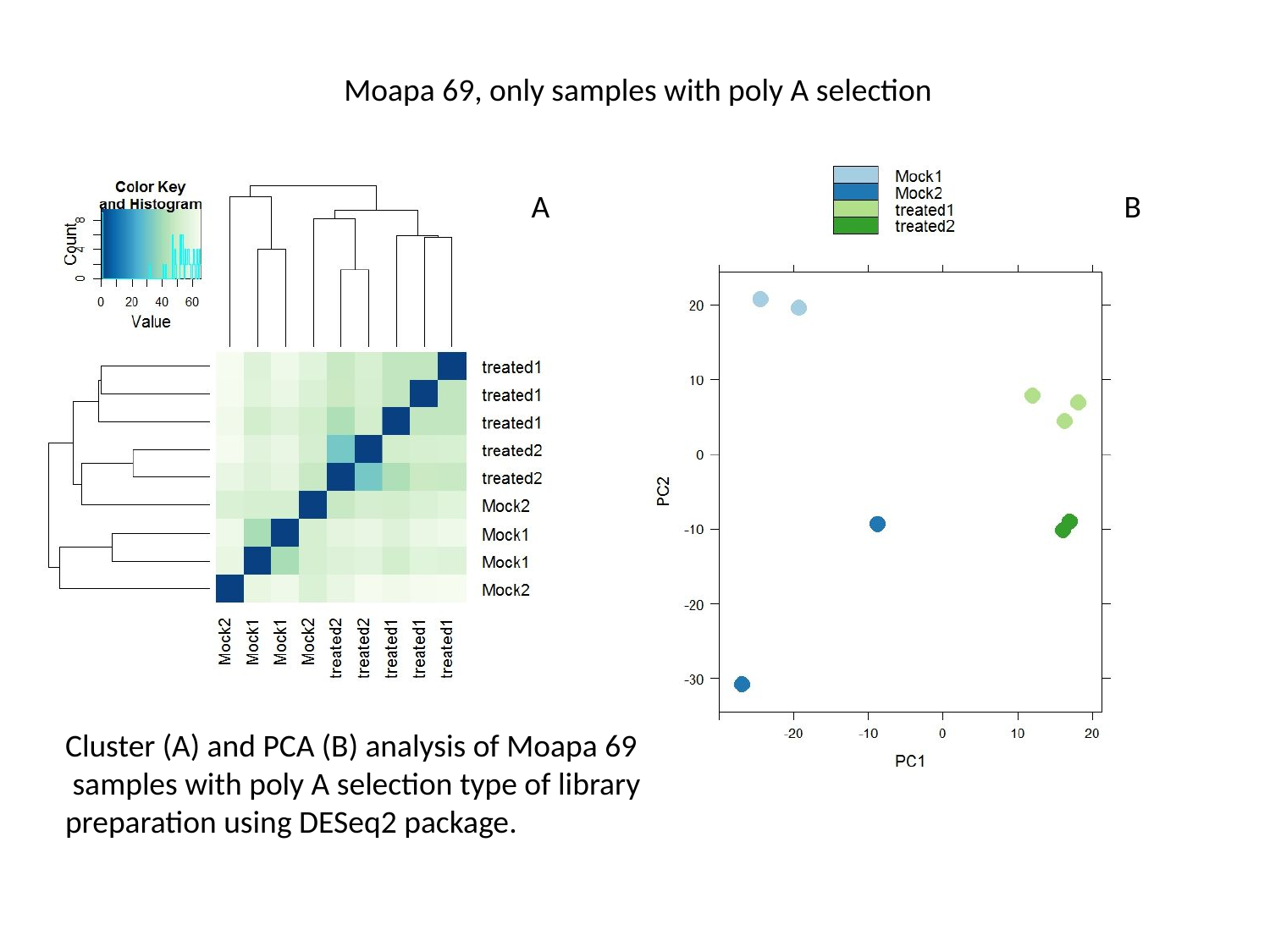

Moapa 69, only samples with poly A selection
A
B
Cluster (A) and PCA (B) analysis of Moapa 69
 samples with poly A selection type of library preparation using DESeq2 package.
