# Supplementary material for: Transcriptome Analysis of Resistant and Susceptible Alfalfa Cultivars Infected With Root-Knot Nematode Meloidogyne incognita
Source: PLoS One. 2015 Feb 24;10(2):e0118269. doi: 10.1371/journal.pone.0118269 (PMC4339843; doi:10.1371/journal.pone.0118269)
Supplement: S2 Fig — (PPTX) [file pone.0118269.s002.pptx]

## Slide 1
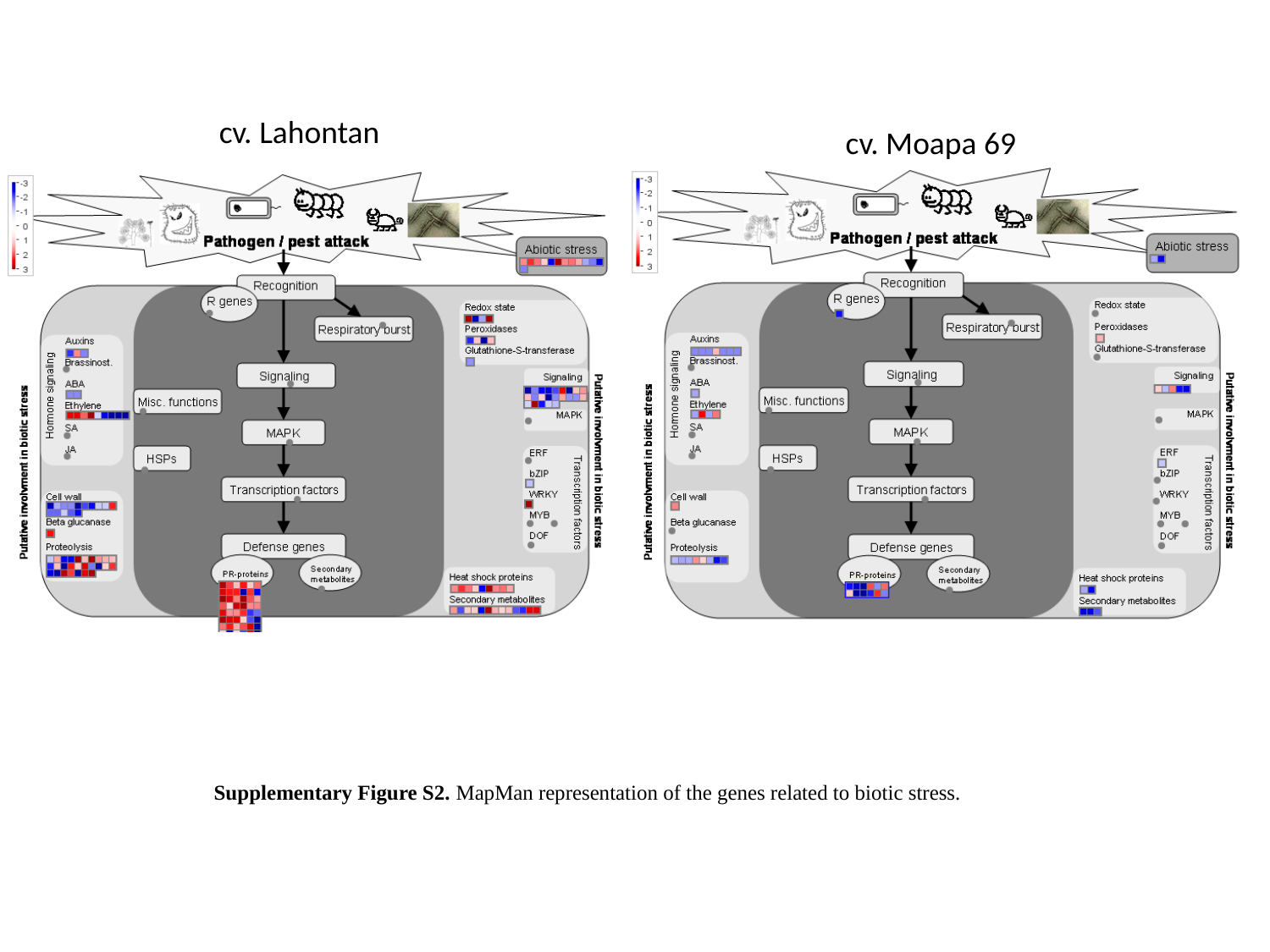

cv. Lahontan
cv. Moapa 69
Supplementary Figure S2. MapMan representation of the genes related to biotic stress.
