# Supplementary figures and images for: Transcriptome Analysis of Resistant and Susceptible Alfalfa Cultivars Infected With Root-Knot Nematode Meloidogyne incognita
Source: PLoS One. 2015 Feb 24;10(2):e0118269. doi: 10.1371/journal.pone.0118269 (PMC4339843; doi:10.1371/journal.pone.0118269)

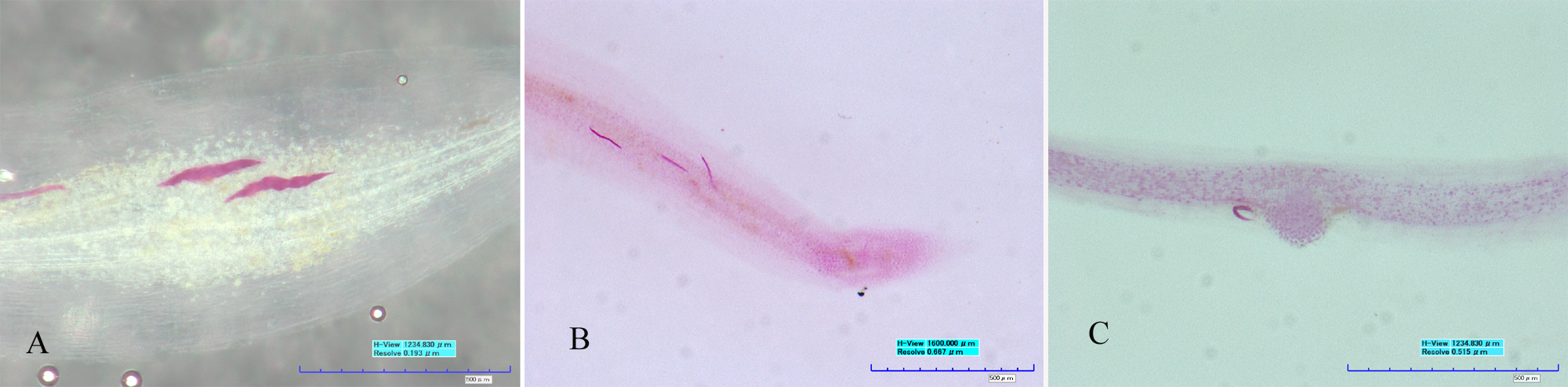

Supplement: S3 Fig — A: cv Lahontan, 7dpi. B: cv Moapa, 72 hrs post inoculation. C: cv Moapa, 7dpi. (TIF) [file pone.0118269.s003.tif]
