# Supplementary material for: Correction: Transcriptome Analysis of Resistant and Susceptible Alfalfa Cultivars Infected With Root-Knot Nematode Meloidogyne incognita
Source: PLoS One. 2015 Mar 30;10(3):e0123157. doi: 10.1371/journal.pone.0123157 (PMC4379156; doi:10.1371/journal.pone.0123157)
Supplement: S2 File — (PDF) [file pone.0123157.s002.pdf]

RESEARCH ARTICLE

Transcriptome Analysis of Resistant and Susceptible Alfalfa Cultivars Infected With Root-Knot Nematode *Meloidogyne incognita*

Olga A. Postnikova[1], Maria Hult[2], Jonathan Shao[1], Andrea Skantar[2], Lev G. Nemchinov[1]*

1 USDA-ARS, Beltsville Agricultural Research Center, Molecular Plant Pathology Laboratory, Beltsville, Maryland, United States of America, **2** USDA-ARS, Beltsville Agricultural Research Center, Nematology Laboratory, Beltsville, Maryland, United States of America

* Lev.Nemchinov@ars.usda.gov

OPEN ACCESS

Citation: Postnikova OA, Hult M, Shao J, Skantar A, Nemchinov LG (2015) Transcriptome Analysis of Resistant and Susceptible Alfalfa Cultivars Infected With Root-Knot Nematode *Meloidogyne incognita*. PLoS ONE 10(2): e0118269. doi:10.1371/journal.pone.0118269

Academic Editor: John Jones, James Hutton Institute, UNITED KINGDOM

Received: September 30, 2014

Accepted: January 12, 2015

Published: February 24, 2015

Copyright: This is an open access article, free of all copyright, and may be freely reproduced, distributed, transmitted, modified, built upon, or otherwise used by anyone for any lawful purpose. The work is made available under the Creative Commons CC0 public domain dedication.

Data Availability Statement: All relevant data are within the paper and its Supporting Information files.

Funding: This work was supported by the United States Department of Agriculture, Agricultural Research Service. The funder had no role in study design, data collection and analysis, decision to publish, or preparation of the manuscript.

Competing Interests: The authors have declared that no competing interests exist.

Abstract

Nematodes are one of the major limiting factors in alfalfa production. Root-knot nematodes (RKN, *Meloidogyne* spp.) are widely distributed and economically important sedentary endoparasites of agricultural crops and they may inflict significant damage to alfalfa fields. As of today, no studies have been published on global gene expression profiling in alfalfa infected with RKN or any other plant parasitic nematode. Very little information is available about molecular mechanisms that contribute to pathogenesis and defense responses in alfalfa against these pests and specifically against RKN. In this work, we performed root transcriptome analysis of resistant (cv. Moapa 69) and susceptible (cv. Lahontan) alfalfa cultivars infected with RKN *Meloidogyne incognita*, widespread root-knot nematode species and a major pest worldwide. A total of 1,701,622,580 pair-end reads were generated on an Illumina Hi-Seq 2000 platform from the roots of both cultivars and assembled into 45,595 and 47,590 transcripts in cvs Moapa 69 and Lahontan, respectively. Bioinformatic analysis revealed a number of common and unique genes that were differentially expressed in susceptible and resistant lines as a result of nematode infection. Although the susceptible cultivar showed a more pronounced defense response to the infection, feeding sites were successfully established in its roots. Characteristically, basal gene expression levels under normal conditions differed between the two cultivars as well, which may confer advantage to one of the genotypes toward resistance to nematodes. Differentially expressed genes were subsequently assigned to known Gene Ontology categories to predict their functional roles and associated biological processes. Real-time PCR validated expression changes in genes arbitrarily selected for experimental confirmation. Candidate genes that contribute to protection against *M. incognita* in alfalfa were proposed and alfalfa-nematode interactions with respect to resistance are discussed.

Introduction

After the arthropods, nematodes are the most populated phylum comprising ~ 80,000 of currently described species and originating about one billion years ago [1]. A large number of nematodes (40% of the described species) are free-living organisms that consume bacteria, fungi, other nematodes and protozoa [2]. Other species are parasites of animals (44% of the described species) and obligate parasites of plants (15% of the described species) [2].

Average worldwide crop losses due to plant-parasitic nematodes constitute 12–14% or more than $100 billion per year [3–5]. Ubiquitous root-knot nematodes (RKN) of the genus *Meloidogyne* are among the most damaging and economically important species [6–8]. As a result of RKN feeding, large galls or "knots" are formed on the roots of infected plants. They consist of giant multinucleate cells that never divide and serve as nutrition reservoirs for nematodes, providing diminished resources for the rest of the plant [4].

Among nearly 100 described species in the genus *Meloidogyne*, four represent a major threat to agricultural crops: *Meloidogyne incognita*, *Meloidogyne arenaria*, *Meloidogyne javanica* and *Meloidogyne hapla* [8]. RKN *M. incognita* is the most common and destructive nematode species found in all agricultural regions worldwide [4] and is considered as "the single most damaging crop pathogen in the world" [9]. According to Commonwealth Agricultural Bureaux International (CABI), it is widespread in 25 states of the USA (CABI Invasive species compendium, http://www.cabi.org/isc/).

Alfalfa (*Medicago sativa*), the most extensively cultivated forage legume and the fourth most widely grown crop in the US [10], is a host for several important nematode species, namely stem nematode (*Ditylenchus dipsaci*), root lesion nematode (*Pratylenchus* spp.), cyst nematode (*Heterodera* spp.) and root-knot nematode (*Meloidogyne* spp.). Five species of RKN are of importance on alfalfa, including the southern RKN *M. incognita*, the northern RKN *Meloidogyne hapla*, the Columbia RKN, *M. chitwoodi*, the Javanese RKN *M. javanica*, and the peanut RKN *M. arenaria*. These nematodes are a significant economic concern to alfalfa production because of their wide distribution, broad host range and the serious damage they can cause to the crops grown in rotation with alfalfa [11, 12]. Although many sources of genetic resistance to RKN have been identified and characterized in other crops [5], little information exists on the mechanisms of resistance responses to RKN in alfalfa. Similarly to many commercially available alfalfa cultivars resistant to other environmental factors, alfalfa plants resistant to RKN are selected from germplasm with field resistance or resistance obtained under experimentally controlled conditions. In most cases, alfalfa-nematode interactions and mechanisms underlying the observed resistance to nematode infection remain uncharacterized and poorly understood at the molecular level.

Whereas early attempts to address this question provided new insights into the basis of alfalfa susceptibility and resistance to RKN [13,14], recently developed genomic resources and technologies that quantify changing expression levels during development and under different conditions [15] have not yet been applied towards improving our understanding of these complex interactions. In the meantime, it was noted that the alfalfa resistance response to RKN lacks the typical hypersensitive reaction (HR) seen in tomato and tobacco [14]. This observation was later confirmed in a model legume, *Medicago truncatula*, a close relative of alfalfa [16]. Thus, it appears that the biological processes that underline resistance to nematodes in alfalfa are quite unique, which may also be reflected in the structure of alfalfa transcriptome.

Here, we approached this problem by means of transcriptome profiling of two alfalfa cultivars contrasting in response to RKN *M. incognita*. Prior to this work, RNA-sequencing had never been used to study alfalfa-nematode interactions even though this radical technology can transform research in the field and open up new possibilities for the investigation of survival strategies employed by both a host plant and a parasitic nematode.

Materials and Methods

Plant material, inoculum preparation and inoculation

Two alfalfa cultivars contrasting in response to *M*. *incognita* were used in the experiments: Moapa 69 (resistant) and Lahontan (susceptible) [13, 17]. For germination on the medium, seeds were scarified with H_2SO_4, surface sterilized with 70% ethanol for 3 min and with 1.2% sodium hypochlorite solution for 10 min, rinsed with distilled water and placed on 1% water agar, pH 5.7. Three-day old seedlings were cut at hypocotyls and the roots were transferred to individual Petri dishes (3 roots per dish) containing Gamborg's B5 medium with vitamins and sucrose supplemented with 0.1% PPM [18, 19].

Excised roots were left to grow until they filled up a significant amount of the media plate and developed lateral roots. Inoculum of *M*. *incognita* was prepared by extracting nematode eggs from infected roots of pepper plants, (cv. PA136), using agitation in 10% household bleach (adaptation of [20]). The eggs were rinsed with distilled water on 500 μm-pore sieves. A population of 800–900 eggs in 1 ml of water was transferred onto excised root cultures, sealed with Parafilm and kept in an incubator at 28°C. Before samples were collected, separate sets of root cultures were observed for a period of one month post inoculation to confirm establishment of nematode infection. Infected roots, stained with acid fuchsine were scanned for the presence of nematodes under the Zeiss light microscope. Conventional PCR with *M*. *incognita*-specific primers (S1 Table) was also used to confirm infection.

For RNA-seq, roots were collected 10 dpi, thoroughly rinsed with distilled water and used for total RNA extraction. Four replicates (one Petri dish with excised roots represented one replicate) were used for the inoculated roots and non-inoculated control.

According to Potenza et al. [13], changes in gene expression in both cultivars were noticeable 24 hrs after inoculation, more pronounced 72 hrs after inoculation, and were not observed 14 dpi. Since we collected roots 10 dpi and it took on average 3 days for J2 nematodes to hatch from eggs, our time point represents 7 dpi.

RNA extraction, first-strand synthesis and quantitative real-time PCR

Total RNA extraction, first-strand synthesis and quantitative real-time PCR (qPCR) were performed essentially as described in Postnikova et al [21]. Amplification was conducted with an iQ SYBR Green Supermix kit (Bio-Rad Laboratories, Inc.) on the MiniOpticon Real-Time PCR system (Bio-Rad) in three to four biological and two technical replicas using the following parameters: 94°C/1 min (one cycle); 94°C/30 s, 60°C/30 s, 72°C /30 s (30 cycles). cDNA for qPCRs was made from the same RNA samples that were used for RNA-sequencing. Previously detected NP_001237047, an unknown gene with little variation in expression levels, was used as a reference in all qPCR experiments [21]. The specificity of all amplifications was confirmed by single-peak melting curves. Delta Delta C (T) method ($2^{-\Delta\Delta C}{}_{T}$) was used for analysis of relative expression. A ratio between each of the nematode-infected samples and a corresponding average of the mock-inoculated samples was calculated. To obtain a final ratio for any given gene, an average and a standard deviation (SD) for all biological replicates were calculated.

RNA-seq, transcriptome assembly and analysis

RNA-sequencing was performed by GENEWIZ, Inc (South Plainfield, NJ, USA) for a fee using the Illumina HiSeq 2000 system. There were four replicates for each experimental condition. For each of the two cultivars, eight samples representing roots from individual Petri dishes were sequenced (four control and four infected). Two types of cDNA libraries were generated (polyA selection and rRNA depletion) and combined for the transcriptome assembly. Only the

polyA selection libraries were used for the expression profiling since the cDNA libraries derived from rRNA depletion method did not form correct clusters (S1 Fig.).

To achieve a high quality alfalfa root transcriptome, *de-novo* transcripts were acquired using a range of *k*-mer sizes (*k*-mers 49, 53, 55 and 57) by Oases 0.2.08 and Velvet 1.2.07 genome assemblers. Since the libraries were obtained by paired-end sequencing, the 'shortPaired' parameter was used in Velvet. The file sizes for each library on average ranged from 4.GB to 6GB, which corresponds to 15 million to 25 million reads for each file, respectively, or 30 million to 50 million reads for each pair. On average, each assembly with varius *k*-mers yielded between 120,000 to 200,000 contigs. In order to generate and improve a complete alfalfa transcriptome and utilize many *de novo* assemblies, these results were processed with the EvidentialGene pipeline, as described in Nakasugi et al. [22]. The process started with CD-HIT program that removes identical fragments, thus reducing the redundancy from combining the assemblies (http://weizhongli-lab.org/cd-hit/). Next, the EvidentialGene pipline extracted biologically meaningful transcripts from the resulting CD-HIT pool of sequences utilizing the coding potential. The EvidentialGene pipeline produces the best coding sequences (CDS and amino acid sequences) among highly similar transcripts though blast alignments. The resultant transcriptome served as a template for gene expression profiling. The DESeq 2 package from Bioconductor [23] was used to estimate sample quality (PCA) and the expression level of transcripts. The DESeq package is a program in the R statistical software suite that accepts raw counts of sequencing reads from high throughput experiments as input into the program. The DEseq program performs normalization, variance estimation and differential expression of the raw read counts and works best with experiments with replicates. Differential expression [>2 fold, false discovery rate (FDR) < 0.025] of alfalfa genes was assessed by mapping reads to the *de novo*-assembled TCs using Bowtie2–2.1.0. Raw counts of the mapped reads were extracted from the Bowtie alignment for each library. After transcriptome assembly, genes were annotated and assigned to known functional groups as previously described [21]. For detection of differentially expressed transcripts in nematodes a publicly available *M*. *incognita* database was used (www.nematode.net).

Results

M. *incognita* on alfalfa root culture

Preliminary experiments demonstrated that *M*. *incognita* grew and reproduced well on alfalfa excised root culture. Primary signs of infection represented by root galls were first visually detected on susceptible cv. Lahontan at 10 days post-inoculation (dpi). In most cases, with few exceptions, galls were not formed on roots of resistant cv. Moapa 69, which has an approximate expected resistance to *M*. *incognita* of 50% [17]. Four weeks after inoculation, infective juveniles and sedentary female nematodes were readily observed on excised roots of cv. Lahontan (Fig. 1A and B); females were often surrounded by laid eggs (Fig. 1C).

Overview of the generated assemblies

A total of 1,701,622,580 short reads were obtained. Seven different transcriptomes were assembled using four *k*-mers values (49, 53, 55 and 57) and then combined into a high quality single transcriptome with the EvidentialGene pipeline [22], (Table 1). We were able to map back to the largest assembly ~ 84% of paired end reads in each individual sample with Bowtie2 Aligner [24]. Between 80% and 81.5% of all assembled tentative consensus sequences (TCs) had significant (e-value < 0.00001) blast hits with the protein database and *Medicago truncatula* database M.t.4.1, respectively (S2A and S2B Tables). Based on the nr database the total assembly contained 25,151 unique genes. This number is close enough to 31,661 high confidence gene

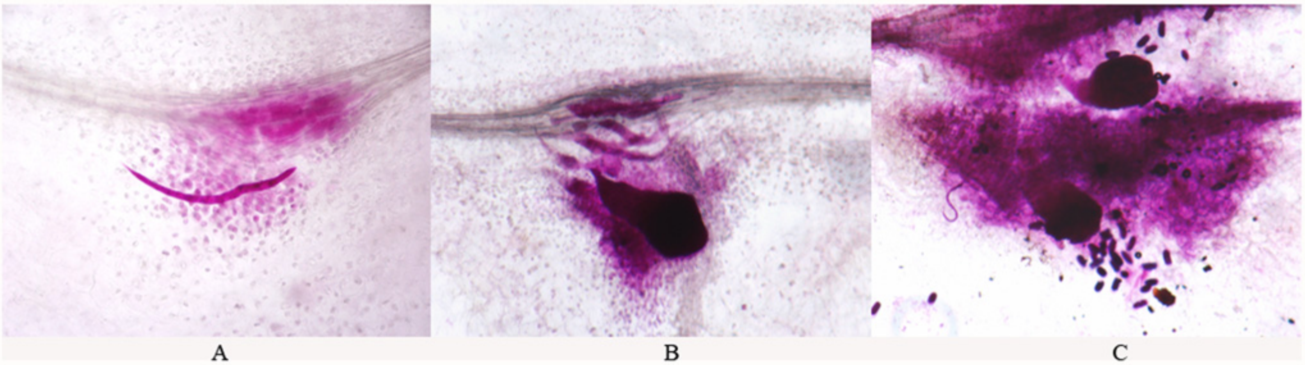

Fig 1. *M. incognita* on alfalfa excised root culture, four weeks post inoculation. A: infective juveniles. **B**: female nematode and infective juveniles. **C**: sedentary females were often surrounded by laid eggs.

doi:10.1371/journal.pone.0118269.g001

models found in *M. truncatula* up to date. Thus, root transcriptome assessment showed that data obtained by RNAseq are sufficient for gene expression profiling (Table 1).

Identification of the nematode-responsive transcripts and their functional categorization

Differential expression [>2 fold, false discovery rate (FDR) <0.025] of alfalfa genes of both cultivars was evaluated using DESeq 2 tool [23]. Count data were obtained by mapping reads to the *de novo*-assembled TCs with Bowtie 2 [24].

Cv. Lahontan. We found 1143 differentially expressed TCs in this susceptible to *M. incognita* cultivar (Table 2). These TCs included unknown sequences with no blast hits. All TCs with sequence similarity to the nematode were removed from the list. Among 1143 TCs, 923 had high similarity scores with *M. truncatula* (e-value <0.00001) and 712 of them were unique (non-redundant). Relative to all non-redundant TCs with sequence similarity to *M. truncatula*, DEG represented 3%. The number of induced and repressed unigenes in cv Lahontan during nematode infection was similar (373 vs 339, respectively; S3 Table).

Using AgriGO software [25], 712 unigenes were further assigned to Gene Ontology (GO) terms for annotation and description of their prospective biological functions. GO overrepresentation analysis showed that GO terms "response to stress", "response to stimulus" and "defense response" in the domain "biological process" were significantly overrepresented among both up- and down-regulated genes (S3 Table). Other categories, such as "regulation of

Table 1. *De-novo* assembly statistics.

Cultivars	Assemblies (*k*-mer values 49,53,55,57)	No of sequenced cDNA libraries	No TCs	Minimum TCs length	Maximum TCs length	Maximum protein length	Mt 4.1 database E-value <(1E-5)	NR database E-value <(1E-5)
Lahontan	Mock	5	37497	123	15666	5221		
	Infected	5	37681	123	15306	5101		
	Combined	10	47590	123	15666	5221	41628	40844
Moapa 69	Mock	5	36631	123	15306	5101		
	Infected	6	36605	123	15306	5101		
	Combined	11	45595	123	15306	5101	40144	39382
	Combined 2 cvs	21	59831	123	15666	5221	48431	48796

doi:10.1371/journal.pone.0118269.t001

Table 2. Differentially expressed transcripts (DETs) found in resistant and susceptible cultivars.

DETs	TCs	Mt-related TCs	Unique TCs	Common
Lahontan	1143	923	712	51
Moapa	319	246	217	51
Basal ratio	2350	1968	1433	

TCs: total number of differentially expressed tentative consensus sequences (TCs)
Mt-related: DETs that were found to be related to *Medicago truncatula* by BLASTp
Unique TCs: non-redundant DETs orthologous to *M*. *truncatula*
Common: DETs differentially expressed in both cultivars
Basal ratio: DETs found under normal conditions, ratio cv. Moapa/cv.Lahontan

doi:10.1371/journal.pone.0118269.t002

cellular process" "biological regulation", "translation", etc were significantly overrepresented only among the up-regulated gene set, suggesting involvement of the corresponding biological processes in the general response of this cultivar to nematode infection. The number of genes in the overrepresented GO categories is shown in Fig. 2.

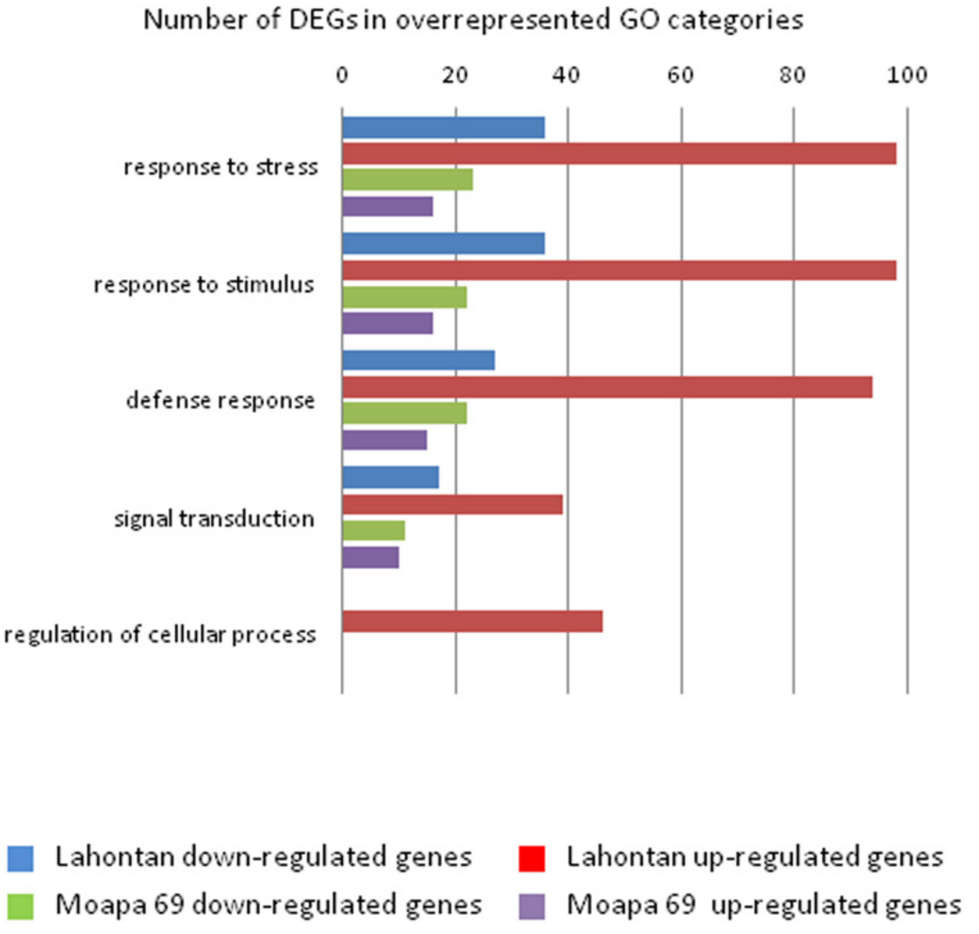

Fig 2. Number of genes in the overrepresented GO categories of cv. Lahontan and cv. Moapa.

doi:10.1371/journal.pone.0118269.g002

Cv. Moapa 69. Transcriptome analysis of the resistant line identified 319 differentially expressed TCs, including unknown and excluding nematode-related sequences (Table 2). This number comprised 246 TCs with sequence similarity to *M. truncatula*, containing 217 differentially expressed individual unigenes. The majority of the DEGs in cv Moapa were down-regulated (135) and the expression of 82 genes was induced (S4 Table). Thus, the number of genes, responsive to nematode infection in the resistant cultivar decreased more than threefold, as compared to the susceptible line. However, most of the 217 unigenes from Moapa 69 were classified into overrepresented GO terms almost exclusively related to defense and signaling functions: "defense response", "response to stress", "response to stimulus", "signaling" etc; (Fig. 2 and S4 Table).

Comparison of the DEGs between susceptible and resistant cultivars. Sets of the DEGs revealed in Lahontan and Moapa 69 were compared with each other. Contrasting the responses of DEGs that are common to both cultivars and distinguishing the set of DEGs that are unique for each line can help to identify genes that may be critical for genetic susceptibility or resistance to *M. incognita*. We found that 51 common genes were differentially expressed in the two cultivars while 827 DEGs were unique to one or the other (Fig. 3 and S5 Table). Among the common genes, 36 and 21 DEGs were up-regulated while 15 and 30 DEGs were down-regulated in Lahontan and Moapa 69, respectively (S5 Table). Expression of some common genes was discordant between the lines; two clusters of these discordantly expressed genes had elevated basal expression levels in the resistant ecotype (S5 Table), suggesting their specific roles in response to the infection.

Interestingly, when the composition of the common and distinct DEGs was evaluated, we found that both sets contain a significant number of genes that are associated with resistance against pathogens (*R* genes), (S5 Table). Putative *R* genes were detected by blastp search against manually curated *R* genes of the PRGdb (112 genes), an open-source database of plant resistant genes [26]. The PRGdb recognizes at least five R protein types: TIR-NBS—LRR, CC-NBS-LRR, RLK (receptor-like kinases), RLP (receptor-like protein) and RLK (kinase-like protein) [26, 27]. However, because many *R* genes are missing from the manually curated PRGdb or not classified as such, we added to the blastp output known, annotated resistant genes from *M. truncatula* (12 genes) and screened the final list against our DEGs. This analysis revealed that almost one-third (16) of the common DEGs are *R* genes. Two of them, orthologous to *M. truncatula*'s Medtr3g056585 and Medtr0277s0020.3, were induced in Moapa 69 and repressed in Lahontan. The distinct DEGs of the susceptible line contained 121 (17%) putative *R*-genes, 95 of them were induced and 26 repressed (S4 Table). The resistant cultivar contained 36 (16%) distinct DE *R*-genes: 14 of them were up-regulated and 22 repressed (S5 Table). Apparently, since cv. Lahontan is a susceptible cultivar, the presence of non-specific DE *R*-genes has no influence on a nematode's ability to cause disease and may be part of a general or passive defense reaction. On the contrary, distinct DE *R*-genes found in Moapa 69, especially 14 induced genes, may contain a candidate gene(s) responsible for the unique resistance interactions between this cultivar and RKN [14, 16]. The same is true for the two common *R*-genes up-regulated in Moapa.

GO analysis of the distinct DEGs in cv. Lahontan revealed other interesting categories, not only terms related to "defense response", are overrepresented as well. Distinct up-regulated genes of cv. Lahontan were also distributed among categories "biological regulation", "translation", "hydrolase activity", "ribonucleoprotein complex", "cytoplasm" etc. (S5 Table). Down-regulated genes, in addition to "defense response" and "signaling" terms, were classified into categories "response to oxidative stress", "response to chemical stimulus", "antioxidant activity", "oxidoreductase activity", "peroxidase activity". These terms were not found among overrepresented GO categories of cv. Moapa. Apparently, induction and repression of genes associated with these biological processes in cv. Lahontan reflect not only host basal defense responses during compatible interaction, but also activities related to the successful establishment of nematode parasitism.

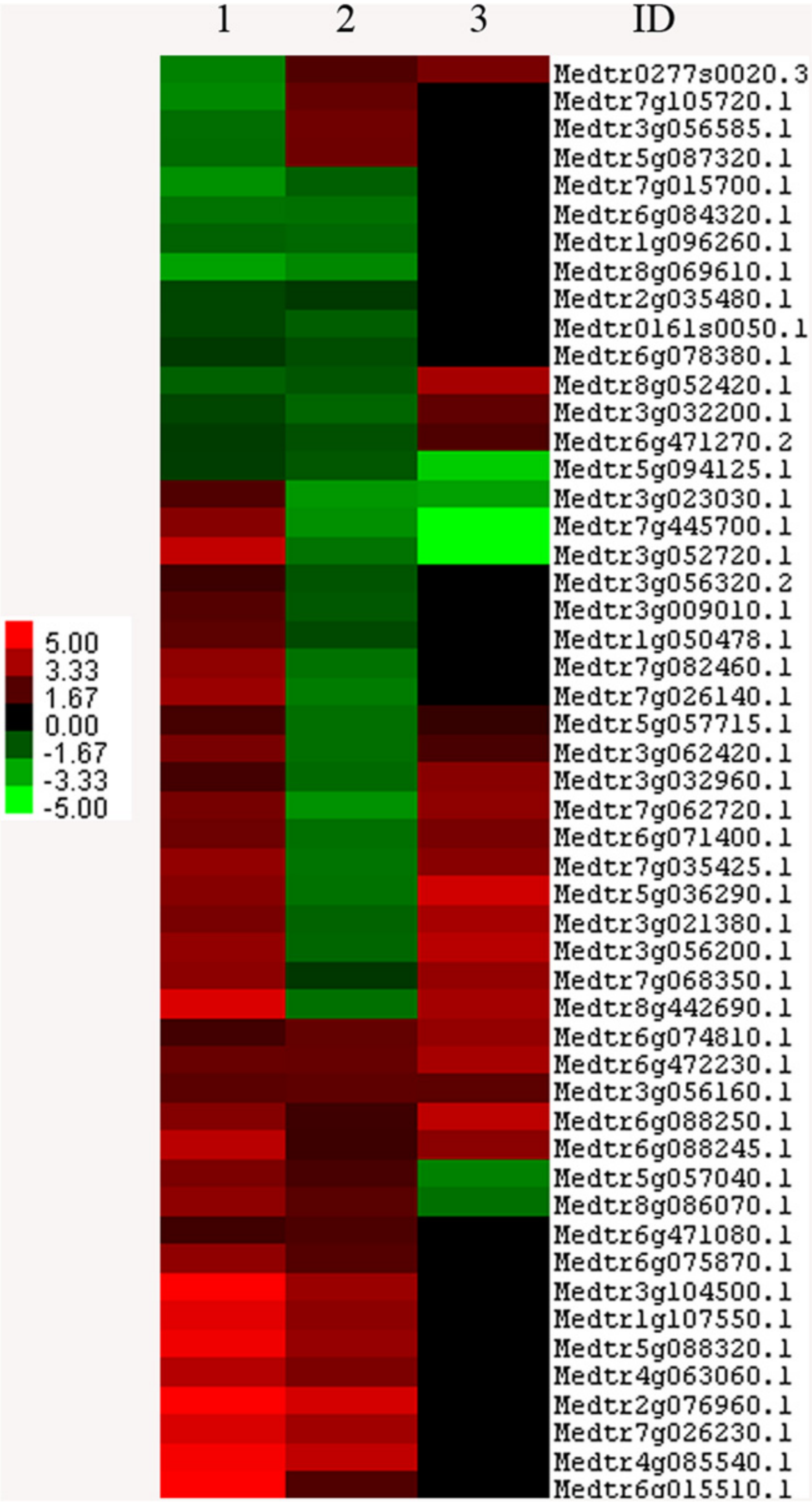

Fig 3. Differentially expressed genes found in both cultivars (common genes). **1**: cv. Lahontan. **2**: cv. Moapa. **3**: basal ratio Moapa/Lahontan. **ID**: *M*. *truncatula* gene ID. Color code is shown to indicate up and-down-regulated genes (red and green, respectively). Clustering was done with Claster.3.0 software [37]. Visualization performed with Java Treeview [38].

doi:10.1371/journal.pone.0118269.g003

Using MapMan annotation software to organize and display our data sets in the context of biological pathways [28], we found 92 genes related to biotic stress responses among distinct DEGs of cv. Lahontan and 17 in cv. Moapa (S2 Fig. and S6 Table). The majority of them are pathogenesis-related proteins (PRs) whose expression varies as much as 36-fold (-4 to > +4, $\log_2$). Production of PRs in response to biotic stress is well-known since most of them possess antimicrobial activity [29]. The presence of significant numbers of induced PRs in the susceptible line (69) as compared to the resistant one (5) may be explained by their involvement in basal defense responses that do not play a critical role in resistance pathways to specific pathogens. DE transcripts associated with ubiquitin dependent proteasome degradation pathway were noticeable in both cultivars, especially in Lahontan, where they were significantly induced. Most intracellular proteins are degraded by this pathway and selectivity of proteolytic processes can indicate different rates of protein synthesis and degradation. In general, a larger variety of MapMan-outlined metabolic processes in the susceptible line suggests a steady, continuous defense response that is overcome by the pathogen.

When we assessed the final transcriptome for the presence of DE transcription factors using TF domain footprints [30], only 24 TFs were located among all DEGs in both cultivars: four in cv. Moapa and 20 in Lahontan (S7 Table). Almost all of them were down-regulated, with the exception of several TFs (4) in cv. Lahontan. No common DEG TFs were detected in both lines. Theoretically, the limited number of DE TFs during infection may be attributed to the temporal difference between transcriptional regulation and changes in gene expression levels.

Basal gene expression levels in normal conditions. Comparing basal gene expression levels in uninfected plants of susceptible and resistant cultivars can provide important clues to the behavior of genes that may influence plant responses to nematode infection. That is, these genes may already be "preconditioned" toward disease, as evident from differences in their basal expression profiles. A total of 1433 DEGs were found when two cultivars were compared to each other under normal conditions (a ratio between Moapa 69/Lahontan, S8 Table). Among them, basal expression of 769 genes was elevated in the resistant line. When these 769 genes were screened against 82 DEGs up-regulated during RKN infection, 13 genes matched, i.e. had concordant expression in healthy and infected plants of cv. Moapa 69 (S8 Table). In this group, there were six *R* genes orthologous to *M. truncatula* Medtr6g088245.1, Medtr6g072450.1, Medtr6g074810.1, Medtr6g088250.1, Medtr0277s0020.3 and Medtr6g472230.1 and one RNA-binding protein with CCCH-type zinc finger domain orthologous to Medtr3g056160.1.

Comparison between 1433 genes differentially expressed under control conditions and 51 common DEGs found in both cultivars during RKN infection revealed 26 DEGs (Fig. 3 and S8 Table). Nine of them were concordant in healthy and infected plants of cv. Moapa 69 (5 induced and 4 repressed). In this cluster, the *R* gene of the TIR-NBS-LRR class, orthologous to Medtr0277s0020.3, was notably up-regulated in healthy and infected roots of Moapa, while its expression was reduced in the susceptible cultivar during RKN infection. Discordant DEGs in cv. Moapa 69 included 16 genes: 14 of them changed their expression pattern from being induced under normal conditions to becoming repressed under infection, and two down-regulated genes became induced during infection. Promising DEGs obtained by basal expression profiling are summarized in S8 Table.

Confirmation of the transcriptomic data by Quantitative Real-time PCR

QPCR was performed with 33 arbitrarily selected unique genes classified as differentially expressed based on the transcriptomic analysis. Most of them were chosen from DEGs common between two lines so that expression could be experimentally validated in both cultivars. QPCR

results showed a strong correlation with transcriptomic data (Pearson correlation coefficients r = 0.7, Table 3). Particularly interesting are two genes that were up-regulated during infection in Moapa 69 and repressed in Lahontan (Table 3). One of the DEGs is likely to be an *R* gene with a characteristic NBS-LRR domain (Medtr3g056585.1) and another DEG (Medtr5g087320) was a receptor-like protein kinase that also has a blast hit with resistant genes.

Identification of *M*. *incognita* transcripts

A publicly available *M*. *incognita* database (www.nematode.net) containing 7,980 transcripts was used to pinpoint nematode TCs in infected root transcriptomes of both cultivars with Bowtie 2 aligner [24]. On average, alignment rates for both lines were 0.4% from the total number of reads obtained. Only 348 *M*. *incognita* transcripts were assembled by more than 50 short reads (S9 Table).

Unfortunately, current genome annotation of *M*. *incognita* (http://www7.inra.fr/meloidogyne_incognita/genomic_resources/downloads) that would enable a comprehensive description of our TCs, is in poor condition: complete annotation tables and GFF files with start and end coordinates, are not accessible for downloading.

For this reason, we attempted to classify nematode-related TCs into GO terms using the annotated genome of *C*. *elegans* (http://www.uniprot.org/uniprot/?query = taxonomy:6239). Since *C*. *elegans* is not a parasitic nematode, this analysis was meant to place genes into general biological pathways and metabolic processes that may not necessarily be involved in parasitism in *M*. *incognita*.

Among the 348 TCs, 208 had blast hits with *C*. *elegans*, 16 with other species and 124 did not have any blast hits (S9 Table). Out of 208 TCs, only 78 were distributed into GO terms based on the similarity with *C*. *elegans* genome (S9 Table). Some potentially interesting or highly expressed *M*. *incognita* TCs, which might be important for parasitic relationship did not have any match with *C*. *elegans*. Two of these genes (*M*. *incognita* TCs BM882635, the C-type lectin-like domain superfamily and TA989_6306, putative NADH-ubiquinone/plastoquinone, complex I protein), along with the gene orthologous to *C*. *elegans* (BM774159, protein of the CD36 superfamily), were down-regulated in nematodes infecting roots of resistant cultivar Moapa 69, as compared to the nematodes from susceptible cv. Lahontan (S9 Table). Three other genes without any blast hits with *C*. *elegans* genome (*M*. *incognita* TCs TA2213_6306, no blast hits, TA189_6306, blast hit with an unknown protein of parasitic roundworm *Ancylostoma ceylanicum* and CF099765, blast hit with a small integral membrane protein 20 of red flour beetle *Tribolium castaneum*), were highly expressed in nematodes infecting both cultivars.

Discussion

As of today, no studies have been published on global gene expression profiling in alfalfa infected with RKN or any other plant parasitic nematode. Very little information is available about molecular mechanisms that contribute to pathogenesis and defense responses in alfalfa against these pests, and specifically against RKN. A few available published reports, however, pointed to the unique resistant pathway implemented in alfalfa against RKN [13, 14, 16]. As the response of resistant alfalfa does not preclude nematode penetration into the plant, it appears to be cardinally different from the mode of resistance to *M*. *incognita* identified in tomato and tobacco due to the lack of localized hypersensitive response (HR). According to Williamson and Kumar [5], nematode resistance, as a general rule, is characterized by a localized programmed cell death at or near the feeding site. This reaction is normally controlled by a single or multiple resistance genes. Pioneering studies of Potenza and co-authors [13, 14] revealed

Table 3. Confirmation of the transctiptomic data (RNA-seq) by Quantitative Real-time PCR (Q-PCR.

	**cv. Lahontan**		
***M. truncatula* ID**	**RNA-seq**	**Q-PCR**	**Description**
**Medtr1g028720.1**	**-2.97**	**-3.14**	**|NB-ARC domain disease resistance protein**
Medtr3g027330.1	1.80	-2.07	|receptor-like protein|
**Medtr5g036290.1**	**2.65**	**1.51**	**|matrixin family protein**
**Medtr6g075870.1**	**2.86**	**1.98**	**|disease resistance protein (TIR-NBS-LRR class)**
**Medtr7g065160.1**	**-3.85**	**-0.38**	**|Lipid transfer protein**
**Medtr1g115135.1**	**-2.32**	**-1.31**	**|calcium-binding EF hand-like protein**
**Medtr3g023030.1**	**1.58**	**0.59**	**|LRR and NB-ARC domain disease resistance protein**
**Medtr1g027290.1**	**2.57**	**1.84**	**|flavonolsynthase/flavanone3-hydroxylase**
**Medtr7g068350.1**	**2.76**	**1.55**	**|transmembrane protein, putative**
Medtr0277s0020.3	-2.54	0.51	|disease resistance protein (TIR-NBS-LRR class)
**Medtr7g105720.1**	**-2.71**	**-1.33**	**|hypothetical protein**
**Medtr3g056585.1**	**-2.17**	**-2.01**	**|LRR and NB-ARC domain disease resistance protein***
**Medtr5g087320.1**	**-2.14**	**-2.05**	**|receptor-like protein***
Medtr3g052720.1	3.82	-0.67	|organelle transcript processing protein, putative
Medtr7g082460.1	2.83	-0.49	|receptor-like kinase
**Medtr7g026140.1**	**3.05**	**1.66**	**|heat shock cognate70kDaprotein**
**Medtr8g015830.1**	**4.90**	**1.15**	**|F-box/FBD-like domain protein, putative**
**Medtr5g081480.1**	**4.18**	**2.41**	**|hypothetical protein**
**Medtr6g015490.1**	**3.59**	**1.41**	**|disease resistance protein (TIR-NBS-LRR class)**
**Medtr4g105540.1**	**-2.78**	**-1.17**	**|cysteine-rich receptor-kinase-like protein**
**Medtr8g007125.1**	**-3.87**	**-4.29**	**|patatin-like phospholipase**
**Medtr1g052470.1**	**-4.64**	**-12.45**	**|transcription factorbHLH122-like protein**
	**cv. Moapa**		
***M. truncatula* ID**	**RNA-seq**	**Q-PCR**	**Description**
**Medtr5g036290.1**	**-2.26**	**-2.70**	**|matrixin family protein**
**Medtr3g023030.1**	**-2.99**	**-1.51**	**|LRR and NB-ARC domain disease resistance protein**
**Medtr6g045087.1**	**-2.36**	**-1.38**	**|glutamatereceptor3.3,putative**
**Medtr0277s0020.3**	**1.61**	**0.57**	**|disease resistance protein (TIR-NBS-LRR class)**
Medtr7g105720.1	1.98	-2.42	|hypothetical protein
**Medtr3g056585.1**	**2.21**	**0.76**	**|LRR and NB-ARC domain disease resistance protein***
**Medtr5g087320.1**	**2.17**	**2.95**	**|receptor-like protein***
**Medtr3g052720.1**	**-2.27**	**-2.55**	**|organelle transcript processing protein, putative**
**Medtr7g082460.1**	**-2.25**	**-0.79**	**|receptor-like kinase**
**Medtr7g026140.1**	**-2.46**	**-2.58**	**|heat shock cognate70kDa protein**
**Medtr8g442690.1**	**-2.22**	**-5.08**	**|endonuclease/exonuclease/phosphatase family protein**
**Medtr7g066260.1**	**3.13**	**1.77**	**|DUF674family protein**
Medtr5g014380.1	3.03	-0.77	|hypothetical protein
**Medtr7g056277.1**	**-3.41**	**-1.29**	**|F-box/RNI/FBD-like domain protein, putative**
**Medtr6g472230.1**	**2.01**	**1.07**	**|disease resistance protein (TIR-NBS-LRR class), putative**
**Medtr6g074810.1**	**1.95**	**2.49**	**|disease resistance protein (TIR-NBS-LRR class)**
**Medtr3g056160.1**	**1.86**	**1.28**	**|RNA-binding domain CCCH-type zinc finger protein**
**Medtr6g071400.1**	**-2.22**	**-2.00**	**Harpininducingprotein1-likeprotein [M. truncatula]**

(*Continued*)

Table 3. (*Continued*)

	cv. Moapa		
***M. truncatula* ID**	**RNA-seq**	**Q-PCR**	**Description**
**Medtr6g072450.1**	**1.86**	**2.17**	**|disease resistance protein (TIR-NBS-LRR class)**

Bold font: gene expression data consistent between RNA-seq and qPCR;

*, two putative *R*-genes up-regulated during infection in cv. Moapa 69 and repressed in cv. Lahontan.

doi:10.1371/journal.pone.0118269.t003

that resistance to *M. incognita* in alfalfa cv. Moapa 69 does not rely on apoptotic cell death but may occur due to the inability of the RKN to enter developing vascular cylinder of the root, instead remaining clumped at the root apex as early as 48–72 hours after inoculation. Using Northern blot and genomic DNA blot analyses, they further identified four genes that are potentially involved in Moapa 69 resistant interactions with *M. incognita*: glycine-rich RNA-binding protein, phosphoenolpyruvate carboxykinase, isoflavone reductase-like protein, and metallothionein-like protein. While screening the world-wide accession collection of *M. truncatula* with several RKNs, Dhandaydham and co-authors [16] found that one variety, DZA045, was resistant to *M. incognita* without exhibiting an HR response. The authors suggested that a single gene may control resistance in DZA045 by a unique pathway. Judging from their observations showing the absence of HR symptoms and poor development of RKN in the roots, this pathway could be similar to the one described by Potenza et al. in alfalfa [14]. Thus, it appears that in those cases plants may not implement an *R* gene-based response, instead using other defensive strategies of a basal nature which depend on different gene products. Alternatively, if host *R*-genes are involved in the process, their action may differ from typical gene-for-gene interplay leading to the HR after pathogen's recognition by a specific *R* gene [5]. One known example of this kind of interaction is "defense, no death" (dnd) phenotypes in *Arabidopsis thaliana*, which exhibit loss of hypersensitive response (HR) cell death without loss of gene-for-gene resistance [31].

In this work, we attempted to shed more light on these questions by performing root transcriptomic analysis of resistant (cv. Moapa 69) and susceptible (cv. Lahontan) alfalfa cultivars infected with RKN *Meloidogyne incognita*. By generating whole transcriptome profiles, Illumina RNA-seq not only permits a precise estimation of gene expression but is also capable of identifying new genes, transcript variants and genetic polymorphisms. It is especially informative for species without annotated genomes, such as alfalfa.

Identification of DEGs in resistant and susceptible cultivars was of primary importance since differential expression of some of the gene products during nematode infection may be a key to the resistance interactions. Differential expression of the genes found in common between the two lines not only points to the genes participating in general response to infection and to the changes initiated by pathogen to establish parasitism, but also demonstrates contrasting behavior of the same gene products under the infection background. The latter could suggest specific biological roles associated with mechanisms of resistance. In this sense, a discordant expression of common genes between the two lines was of particular interest. In total, we have found 23 common discordantly expressed DEGs (S5 Table). The majority of them (19) were down-regulated in the resistant cultivar. Moreover, 11 of those genes were induced in Moapa in healthy plants, indicating that their declining levels in infected plants are not coincidental. Four common genes, up-regulated during infection in Moapa and repressed in cv. Lahontan, (Medtr3g056585, Medtr5g087320.1, Medtr0277s0020.3, and Medtr7g105720.1), in our opinion, may be of special importance to the resistance pathway in cv. Moapa 69. Presence

of at least two *R* genes among them (Medtr3g056585, an LRR and NB-ARC domain disease resistance protein and Medtr0277s0020.3, a disease resistance protein of TIR-NBS-LRR class) support the idea of atypical gene-for-gene interactions. Furthermore, among 936 distinct DEGs found in both cultivars, 154 (16%) were putative *R* genes, classified as such according to the PRGdb. [26, 27]. Considering that genome of *M*. *truncatula* (45,888 protein-coding transcripts, Mt3.5 v4) contains approximately 2,084 (4.5%) putative *R* genes (predicted from Phytozome, http://www.phytozome.net/), this is an impressive number. Although the level of *R*-genes expression is usually low [5], individual *R* genes may be induced in response to nematode infection and this induction can play a role in signal transduction pathways, leading to resistance [5]. When we examined all DE *R* genes detected in both lines using PDGdb, five of them were found to be involved in nematode resistance: Medtr6g015510.1 and Medtr6g088250.1 in Lahontan and Medtr6g015510.1, Medtr6g088250.1, Medtr6g087200.1, Medtr4g080777.1 and Medtr6g084360.1 in Moapa. All these *R* genes, representing different transcripts, mapped to the same ortholog in *M*. *truncatula*: *Solanum tuberosum* nematode resistance protein (Gro1–4) gene, conferring resistance to pathotype Ro1 of the root cyst nematode *Globodera rostochiensis* [32]. We were not able to locate any information on the occurring of the HR-like phenotype in potato cultivars resistant to *G*. *rostochiensis* pathotype Ro1. Apparently, two *R* genes induced in cv. Lahontan, (orthologous to Medtr6g088250.1 and Medtr6g015510.1) are irrelevant for resistant pathways. However, both of them are up-regulated in cv. Moapa and one *R*-gene, Medtr6g088250.1 is induced in the resistant line during normal conditions. In other words, the level of the Medtr6g088250.1 transcripts, being already significantly elevated in healthy plants as compared to cv. Lahontan, is even more induced after nematode infection. Four genes previously found to be induced in both cultivars during infection with *M*. *incognita* [14] were not among the set of DEGs detected in this work. One possible explanation is the timeline of the experiment: early response to infection [14] vs 7 dpi (this study).

To search for similarities between our data and plant-RKN reactions in other species, we compared them with two published reports: a study on comprehensive gene expression profiling in tomato resistant to RKN [33] and transcription profiling of soybean-root-knot nematode interaction [34]. Responses to RKN in other species varied. Gene expression profiling of tomato resistant to RKN (resistant gene *Mi*-1) revealed 58 differentially expressed genes in resistant roots. Among them only one common gene, orthologous to Medtr3g054080.1 (Solyc11g072630.1.1), was up-regulated both in resistant alfalfa cultivar and in resistant tomato in response to the RKN. In *M*. *truncatula*, it encodes cyclin-dependent (CDK)-activating kinase and in tomato mitogen-activated protein kinase (MAPK). CDKs and MAPKs are related and regulate a variety of cellular functions, such as cell cycle and proliferation, transcription, mRNA processing, response to environmental stimuli etc. Based on alfalfa transcriptome analysis presented in this study, the gene (ID evgevgevgvelvLoc27212t3, S2A and S2B Table) was annotated as a cyclin-dependent kinase.

Comparison of our data with the expression profiling of soybean PI 595099 (resistant line)-RKN pathosystem [34] by direct blast search, revealed 16 common differentially expressed genes in resistant cv Moapa 69 and RKN-resistant soybean line (S10 Table). Interestingly, that two orthologous genes not particularly mentioned by the Beneventi and co-authors [34] as playing a role in resistant reaction, are on our list of gene-candidates involved in resistant response in cv. Moapa (Medtr3g054080.1 and Medtr2g096970.1). One of them is again CDK (Medtr3g054080.1). Both genes were up-regulated in cv Moapa and resistant soybean line. Activation of CDKs and/or MAPKs in resistant lines of three different species in response to *M*. *incognita* may indicate their involvement in molecular mechanisms controlling resistance to root-knot nematode in legumes.

Only 24 TFs were found to be differentially expressed in both lines during nematode infection (S7 Table). Speculatively, the limited number of DE TFs as well as a general decrease in their activity may be attributed to the temporal difference between transcriptional regulation and changes in gene expression levels. On the other hand, down-regulation of this small group of TFs can be a specific requirement for their functional roles in plant defense mechanisms or otherwise in establishment of parasitism.

Little is known about molecular mechanisms of RKN parasitism in alfalfa and nematode genes involved in interaction with this plant host. This question was not a focus of this study; but, since we needed to clearly differentiate nematode-originated sequences from alfalfa transcripts, it created an opportunity to look at the main biological processes involved in parasitism. We were able to find common GO terms corresponding to biological pathways responsive to interactions of nematodes with resistant and susceptible hosts (S9 Table). *M. incognita* genes orthologous to the C-type lectin-like domain superfamily, NADH-ubiquinone/plastoquinone (complex I) protein and CD36 protein family were down-regulated in nematodes infecting resistant cv Moapa. The Metazoan proteins with C-type lectin-like domains (CTLDs) have diverse functions including cell adhesion, intracellular processes and trafficking of proteins [35]. NADH-ubiquinone/plastoquinone (complex I) protein is an enzyme of the respiratory chain located in the mitochondrion and involved in proteasome core complex assembly, response to misfolded protein and ubiquitin-dependent protein catabolic processes (http://www.arabidopsis.org/servlets/TairObject?id=27598&type = locus). CD36 ortholog in *C. elegans* hypothetically acts as a receptor for microbe-derived ligands and is thought to be involved in host resistance to fungal pathogens [36]. Whether reduced activity of these genes in *M. incognita* is associated with failure of the nematode to infect resistant cultivar remains a question (S9 Table).

In brief, transcriptome analysis of two alfalfa cultivars contrasting in resistance to RKN *M. incognita*, identified nearly a thousand DEGs that are presumably involved in basal defense responses (cv. Lahontan) and in resistance pathways (cv. Moapa). Comparison of DEGs between the cultivars revealed a number of transcripts potentially associated with resistance to nematode in cv. Moapa. Their prospective roles in resistance mechanisms were based on analysis of the specific expression levels of DEGs in common and distinct to each of the lines and also on the extensive functional annotations generated by blast, GO and MapMan tools. The most promising gene-candidates are presented in S11 Table.

Conclusions

The results of this study demonstrate that the host response to RKN *M. incognita* is significantly larger in the susceptible cultivar, with the number of DEGs in cv. Lahontan exceeding those from cv. Moapa by three-to-one margin. The same was true for the number of induced transcripts. Apparently, this is related to the fact that *M. incognita* established successful infection in cv. Lahontan while in the resistant line the parasitic interaction with the host was mostly aborted. Potenza and co-authors [13, 14] reported that J2 nematodes were clumped into root tips of cv. Moapa as early as 72 hrs after inoculation but migrated upward into the developing vascular cylinder in the roots of cv. Lahontan. This observation may indicate that most of the changes in plant gene expression critical to the initiation of resistance pathways occurred before this stage. However, the authors [13] were able to detect differential expression in cv. Moapa for as long as two weeks after inoculation. Besides, in our experiment, non-clustered migrating nematodes were occasionally observed in roots of cv Moapa for as long 7 dpi (S3 Fig.), which in addition to the sporadic presence of susceptible genotypes and delayed root penetration by J2 nematodes, could be attributed to the possible mode of resistance different

from the one suggested by Potenza and co-authors [13]. Accordingly, DEGs found in this work could be actively involved in conferring resistance against *M*. *incognita*. Additional transcription profiling at the single-cell level with individually-infected root exudates will provide further answers to these questions.

The raw data and transcriptome assembly obtained in this study were deposited in the Sequence Read Archive, NCBI (BioProject ID PRJNA266116).

Supporting Information

S1 Fig. Comparison between results obtained from polyA selection and rRNA depletion cDNA libraries. Slide 1. Cluster (A, B) and principal component analyses (C, D) of polyA selection and rRNA depletion cDNA library preparation methods using DESeq2 package. Cv. Lahontan (A, C); cv. Moapa 69 (B, D). **Slide 2**. Cluster (A) and PCA (B) analysis of Moapa 69 samples with poly A selection type of library preparation using DESeq2 package.
(PPTX)

S2 Fig. MapMan representation of the genes related to biotic stress.
(PPTX)

S3 Fig. Nematode-infected alfalfa roots of both cultivars stained at various time points. A: cv Lahontan, 7dpi. **B**: cv Moapa, 72 hrs post inoculation. **C**: cv Moapa, 7dpi.
(TIF)

S1 Table. Primers used in conventional and quantitative real-time PCR.
(XLSX)

S2 Table. A. Blast hits with the protein database. B. Blast hits with *Medicago truncatula* database M.t.4.1.
(XLSX)

S3 Table. Differentially expressed genes in cv. Lahontan.
(XLSX)

S4 Table. Differentially expressed genes in cv. Moapa.
(XLSX)

S5 Table. Comparison of DEGs found in susceptible and resistant cultivars.
(XLSX)

S6 Table. MapMan annotations.
(XLSX)

S7 Table. Differentially expressed transcription factors.
(XLSX)

S8 Table. Basal gene expression levels.
(XLSX)

S9 Table. *M*. *incognita* transcripts.
(XLSX)

S10 Table. Common DEGs found in resistant alfalfa, soybean and tomato in response to root-knot nematode *M*. *incognita*.
(XLSX)

S11 Table. Differentially expressed genes-candidates, potentially associated with resistance to nematode in cv. Moapa
(XLSX)

Acknowledgments

We thank Dr. G.R. Buchan for his help with digital video microscope.

Author Contributions

Conceived and designed the experiments: OAP LGN. Performed the experiments: OAP MH LGN. Analyzed the data: OAP JS LGN. Wrote the paper: LGN OAP MH AS.

References

1. Wang DY-C, Kumar S, Hedges BS (1999) Divergence time estimates for the early history of animal phyla and the origin of plants, animals and fungi. Proceedings of the Royal Society of London, 266:163–171. PMID: 10097391
2. Lambert K, Bekal S (2002) Introduction to Plant-Parasitic Nematodes. The Plant Health Instructor. doi: 10.1094/PHI-I-2002-1218-01 Revised 2009 by the Education Center Editorial Board.
3. Sasser JN, Freckman DW (1987) A world perspective on Nematology: the role of the society. Pp 7–14 in Veech J.A. and Dickson D.W. (eds) Vistas on Nematology. Society of Nematologists, Hyattsville, Maryland. 509p.
4. Mitkowski NA, Abawi GS (2003) Root-knot nematodes. The Plant Health Instructor. doi: 10.1094/PHI-I-2003-0917-01 *Revised 2011*.
5. Williamson VM, Kumar A (2006) Nematode resistance in plants: the battle underground. Trends Genet 22:396–403. PMID: 16723170
6. Abad P, Gouzy J, Aury JM, Castagnone-Sereno P, Danchin EGJ, et al. (2008) Genome sequence of the metazoan plant-parasitic nematode *Meloidogyne incognita*. Nat Biotechnol 8:909–15. doi: 10.1038/nbt.1482 PMID: 18660804
7. Williamson VM, Gleason CA (2003) Plant-nematode interactions. Curr Opin Plant Biol 6:327–33. PMID: 12873526
8. Castagnone-Sereno P, Danchin EG, Perfus-Barbeoch L, Abad P (2013) Diversity and evolution of root-knot nematodes, genus Meloidogyne: new insights from the genomic era. Annu Rev Phytopathol 51: 203–20. doi: 10.1146/annurev-phyto-082712-102300 PMID: 23682915
9. Trudgill DL, Blok VC (2001) Apomictic, polyphagous root-knot nematodes: exceptionally successful and damaging biotrophic root pathogens. Annu Rev Phytopathol 39:53–77. PMID: 11701859
10. Li X, Brummer EC (2012) Applied genetics and genomics in alfalfa breeding. Agronomy, 2:40–61.
11. Hafez SL, Sundararaj P (2009) Nematodes of alfalfa and their management (In: Proceedings, 2009 Western Alfalfa & Forage Conference, December 2–4, 2009, Reno, Nevada).
12. Griffin GD (1990) Diseases caused by nematodes. Pp. 46–51 in: Compendium of Alfalfa Diseases, second edition. Stuteville D.L. and Erwin D.C., (eds). APS Press. St. Paul, MN. 84 pp.
13. Potenza CL, Thomas SH, Higgins EA, Sengupta-Gopalan C (1996) Early root response to *Meloidogyne incognita* in resistant and susceptible alfalfa cultivars. J Nematol 28: 475–484. PMID: 19277165
14. Potenza C, Thomas SH, Sengupta-Gopalan C (2001) Genes induced during early response to Meloidogyne incognita in roots of resistant and susceptible alfalfa cultivars. Plant Sci 161: 289–299. PMID: 11448760
15. Wang Z, Gerstein M, Snyder M (2009) RNA-Seq: a revolutionary tool for transcriptomics. Nat Rev Genet 10:57–63. doi: 10.1038/nrg2484 PMID: 19015660
16. Dhandaydham M, Charles L, Zhu H, Starr JL, Huguet T, Cook DR, et al. (2008) Characterization of Root-Knot Nematode Resistance in *Medicago truncatula*. J Nematol 40: 46–54. PMID: 19259519
17. Griffin GD, Peaden RN, Knipe WJ (1991) Root-knot nematode resistance. In: Standard tests to characterize alfalfa cultivars, third edition (2004). North American Alfalfa Improvement Conference, https://www.naaic.org/resource/stdtests.php.
18. Gamborg OL, Murashige T, ThorpeVasil IK(1976) Plant tissue culture media. In Vitro 12: 473–478. PMID: 965014

19. Supramana S, Lewis SA, Mueller JD, Fortnum BA, Ballard RE (2002) Excised root culture for mass production of *Hoplolaimus columbus* Sher (Nemata: Tylenchida). Nematropica 3:5–11.
20. Hussey RS, Barker KR (1973) A comparison of methods of collecting inocula of *Meloidogyne spp*., including a new technique. Plant Disease Reporter, 57:1025–1028.
21. Postnikova OA, Shao J, Nemchinov LG (2013) Analysis of the alfalfa root transcriptome in response to salinity stress. Plant Cell Physiol 54: 1041–55. doi: 10.1093/pcp/pct056 PMID: 23592587
22. Nakasugi K, Crowhurst R, Bally J, Waterhouse P (2014) Combining transcriptome assemblies from multiple de novo assemblers in the allo-tetraploid plant *Nicotiana benthamiana*. PLoS One. 9:e91776. doi: 10.1371/journal.pone.0091776 PMID: 24614631
23. Anders S, Huber W (2010) Differential expression analysis for sequence count data. Genome Biol 11:R106. doi: 10.1186/gb-2010-11-10-r106 PMID: 20979621
24. Langmead B, Salzberg S (2012) Fast gapped-read alignment with Bowtie 2. Nature Methods, 9:357–359. doi: 10.1038/nmeth.1923 PMID: 22388286
25. Du Z, Zhou X, Ling Y, Zhang Z, Su Z (2010) AgriGO: a GO analysis toolkit for the agricultural community. Nucleic Acids Res 38: W64–W70. doi: 10.1093/nar/gkq310 PMID: 20435677
26. Sanseverino W, Roma G, De Simone M, Faino L, Melito S, Stupka E, et al. (2010) PRGdb: a bioinformatics platform for plant resistance gene analysis. Nucleic Acids Res 38 (Database issue):D814–21. doi: 10.1093/nar/gkp978 PMID: 19906694
27. Sanseverino W, Ercolano MR (2012) *In silico* approach to predict candidate R proteins and to define their domain architecture. BMC Res Notes 5:678. doi: 10.1186/1756-0500-5-678 PMID: 23216678
28. Thimm O, Blasing O, Gibon Y, Nagel A, Meyer S, Kruger P, et al. (2004) MAPMAN: a user-driven tool to display genomics data sets onto diagrams of metabolic pathways and other biological processes. Plant J 37: 914–939. PMID: 14996223
29. Van Loon LC, Rep M, Pieterse CM (2006) Significance of inducible defense-related proteins in infected plants. Annu Rev Phytopatho 44:135–62. PMID: 16602946
30. Postnikova OA, Shao J, Nemchinov LG (2014) *In silico* identification of transcription factors in Medicago sativa using available transcriptomic resources. Mol Genet Genomics 289:457–68. doi: 10.1007/s00438-014-0823-7 PMID: 24556904
31. Jurkowski GI, Smith RK Jr, Yu IC, Ham JH, Sharma SB, Klessig DF, et al. (2004) Arabidopsis DND2, a second cyclic nucleotide-gated ion channel gene for which mutation causes the “defense, no death” phenotype. Mol Plant Microbe Interact 17:511–20. PMID: 15141955
32. Paal J, Henselewski H, Muth J, Meksem K, Menéndez CM, Salamini F, et al. (2004) Molecular cloning of the potato Gro1–4 gene conferring resistance to pathotype Ro1 of the root cyst nematode Globodera rostochiensis, based on a candidate gene approach. Plant J 38: 285–97. PMID: 15078331
33. Schaff JE, Nielsen DM, Smith CP, Scholl EH, Bird DM (2007) Comprehensive transcriptome profiling in tomato reveals a role for glycosyltransferase in Mi-mediated nematode resistance. Plant Physiol 144:1079–92. PMID: 17434994
34. Beneventi MA, da Silva OB Jr, de Sá ME, Firmino AA, de Amorim RM, Albuquerque EV, et al. (2013) Transcription profile of soybean-root-knot nematode interaction reveals a key role of phythormones in the resistance reaction. BMC Genomics 14:322. doi: 10.1186/1471-2164-14-322 PMID: 23663436
35. Zelensky AN, Gready JE (2005) The C-type lectin-like domain superfamily. FEBS J. 272:6179–217. PMID: 16336259
36. Rosso MN, Pujol N, Ewbank JJ (2013) Innate immunity in *Caenorhabditis elegans* and other nematodes. In: Kennedy MW, Harnett W, editors. Parasitic nematodes: molecular biology, biochemistry and immunology. CABI Publishing, NY, USA pp 42–66.
37. de Hoon MJL, Imoto S, Nolan J, Miyano S (2004) Open Source Clustering Software. Bioinformatics 20: 1453–1454 PMID: 14871861
38. Saldanha AJ (2004) Java Treeview—extensible visualization of microarray data Bioinformatics 20: 3246–3248. doi: 10.1186/1751-0473-6-3 PMID: 21241501
